# Development of a Dairy Product Enriched With Oleaster Flour: A Novel Gluten‐Free Functional Pudding

**DOI:** 10.1002/fsn3.72282

**Published:** 2026-08-31

**Authors:** İrem Uzunsoy, Fundagül Erem, Elif Ayşe Anli

**Affiliations:** ^1^ Çaycuma Vocational School of Food and Agriculture Zonguldak Bülent Ecevit University Zonguldak Türkiye; ^2^ Department of Food Engineering, Faculty of Engineering Zonguldak Bülent Ecevit University Zonguldak Türkiye; ^3^ Department of Dairy Technology, Faculty of Agriculture Ankara University Ankara Türkiye

**Keywords:** antioxidant, *Elaeagnus angustifolia* L., gluten‐free, pudding

## Abstract

This study aimed to characterize the physicochemical, textural, microbiological, bioactive, and sensory properties of milk‐based puddings enriched with oleaster flour derived from different parts of the oleaster fruit. Oleaster flesh flour (OFF) and oleaster peel flour (OPF) were added to the puddings at a concentration of 5%, and 1% of sodium alginate was used as a gelling agent. Two control puddings prepared without (C1) and with (C2) sodium alginate, and OFF‐containing (OF) and OPF‐containing (OP) puddings were analyzed on the first and tenth days of cold storage. Results showed that adding OFF and OPF slightly increased the pH, titratable acidity, and ash content of puddings, while significantly decreasing the syneresis. OF and OP had approximately 3‐fold higher total dietary fiber content compared to C1 (*p* < 0.05). Color parameters indicated that puddings became darker, redder, and less yellow with oleaster flour. Oleaster‐enriched puddings were firmer and had higher consistency, cohesiveness, and viscosity index values compared to controls. OFF was found to be more effective in increasing the total phenolic content and antioxidant activity of puddings, showing 1.48‐ and 4.54‐fold higher values than C1, respectively. Protocatechuic, chlorogenic, and vanillic acids were identified as the dominant phenolics in oleaster‐enriched puddings. No mold or yeast growth was observed in the puddings. Total mesophilic aerobic bacteria counts were higher in OF and OP; however, the values were within acceptable limits. Although C1 was more preferred sensorily, the scores of the oleaster‐enriched puddings were also above the average.

## Graphical abstract

Oleaster peel and flesh flour have been found to be potential ingredients for puddings. Oleaster flour enrichment led to an improvement in pudding properties, adding value to the final product.

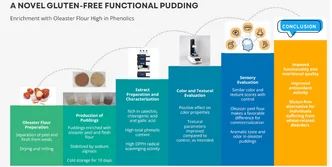

## 1 Introduction

The remarkable relationship between health and functional foods affects consumers to pay more attention to healthy diets. Consequently, this leads to studies investigating food enrichment with functional ingredients possessing health benefits. These functional ingredients cover a large variety of food components such as phenolics, dietary fiber, probiotics, prebiotics, essential fatty acids, vitamins, and minerals, which are found in significant amounts in dairy products and fruits, and demonstrate antioxidant, antimicrobial, anti‐inflammatory, anticancer, and immunomodulating activities (Bagheri et al. 2026; Munekata et al. 2023; Shashirekha et al. 2015).

Milk‐based desserts outshine as appropriate vehicles for delivery of functional ingredients, since they already have a worldwide consumption capacity and a growing market day after day (Şanlı 2023). One of the most consumed and preferred types among all age groups is pudding, due to its nutritional and sensory characteristics (Zarzycki et al. 2019). It is usually prepared by addition of some of the ingredients like wheat flour, starch, egg, sugar, sweeteners, thickeners, flavorings, and fruits which value the product's nutritional and sensory quality (Choobkar et al. 2022). However, wheat flour as the most conventional ingredient used poses a challenge for individuals suffering from wheat‐related disorders such as celiac disease and wheat allergy, with an approximate global prevalence of 1% (Singh et al. 2018). Therefore, it is important to develop alternative gluten‐free pudding types for the mentioned consumers.

Oleaster ( *Elaeagnus angustifolia* L.) is a versatile edible fruit that can be utilized in various industries, including food, pharmaceuticals, cosmetics, and wood (Sharifian‐Nejad and Shekarchizadeh 2019). Oleaster fruit is nutritionally rich, and it can be used as a functional food ingredient owing to its bioactive compounds, dietary fiber, and antioxidant, antimicrobial, and anti‐inflammatory activities (Düşkün et al. 2025; Simsek and Sufer 2021). Therefore, it can be valued as a special ingredient to satisfy the expectations of consumers who want to choose foods that provide positive health effects (Gül et al. 2023). Oleaster flour has been characterized for its functional properties (Sahan et al. 2015) and phenolic compound profile (Karkar and Şahin 2022) earlier, and used as an ingredient in cookies (Sahan et al. 2013, 2019; Şahin 2023a), ice cream (Çakmakçi et al. 2015), doughnut (Sarraf et al. 2017), yogurt (Öztürk et al. 2018), sponge cakes (Kouhanestani et al. 2019; Madadi et al. 2024; Zangeneh et al. 2021), biscuits (Heidari et al. 2022; Incedayi and Erol 2023), bread (Amiri and Aghakeshipour 2023; Ghadarloo et al. 2023; Lavini et al. 2022; Yavuz et al. 2022), breakfast cereals (Tatari et al. 2022), kefir (Gül et al. 2023), mayonnaise (Ünver et al. 2023), crackers (Düşkün et al. 2025), and tarhana (Şahin et al. 2024), particularly due to its rich dietary fiber, phenolic compound content, and antioxidant activity besides the physicochemical, rheological, textural, and structural properties it could impart. To the best of our knowledge, no study has evaluated the potential usage of oleaster flour in puddings. Corn or rice starch are widely used in gluten‐free pudding production. However, it is well‐known that the oleaster has superior bioactive properties compared to corn or rice starch. Then, using oleaster as a raw material in gluten‐free pudding production may lead to a clean‐label, functional pudding alternative. Furthermore, due to the moderate processing temperatures, pudding may serve as a better carrier of bioactive compounds than the solid matrices, such as breads and cookies, which are exposed to higher temperatures during processing. Additionally, other milk‐based products like yogurt, kefir, and ice cream are naturally gluten‐free; therefore, pudding, which can be produced both with and without gluten‐containing ingredients, could be a good option for developing a gluten‐free product. However, formulating a stable pudding with fruit flours is generally challenging. Because most of the fruits have low starch content, which exhibits thickening capacity and a key role in controlling rheological characteristics of the product (Mohamed et al. 2026). Therefore, sodium alginate was incorporated into the formulations to achieve a stable gel network (Chandan and Kilara 2016) and explore its potential to stabilize bioactive compounds during storage. Sodium alginate was preferred, as it does not alter the sensory properties of the foods, is stable against heat treatment, and nontoxic (Biney et al. 2025). The study aimed to assess the impact of adding oleaster peel and flesh flour to milk‐based gluten‐free puddings, by comparing them against wheat flour‐based control groups (gluten‐containing counterparts) with and without sodium alginate. This comparative design may allow for a systematic evaluation of how the ingredients can serve as a novel gluten‐free product with enhanced antioxidant activity. The products were analyzed for their physicochemical, microbiological, textural, and sensory properties during the first and tenth days of cold storage. Additionally, the phenolic composition and antioxidant activity of the samples were also evaluated to determine bioactive properties.

## 2 Materials and Methods

### 2.1 Materials

Partially dried oleasters, whole UHT cow's milk (Migros, Türkiye), wheat flour (Söke, Türkiye), and refined sugar (Bor Şeker, Türkiye) were purchased from a local market. Sodium alginate, used as a gelling agent, was obtained from Smart Chemistry (Türkiye). All chemicals were provided by Merck KGaA (Darmstadt, Germany) unless stated otherwise.

### 2.2 Preparation of OPF and OFF

The peel, flesh, and seeds of oleasters were separated manually. The peel and flesh were dried overnight by a forced‐air incubator (Koçintok, Türkiye) at 40°C and then milled using a grinder (Miza TB200, China). They were passed through a sieve with an aperture size of 500 μm and dried at 40°C until reaching a moisture level below 5%. The samples were packed in zip‐locked polyethylene bags and maintained at 4°C during the study.

### 2.3 Experimental Design

Viscosity is one of the most important quality criteria that directly affects the purchasing behavior for puddings. Therefore, a targeted viscosity range for the experimental pudding samples was determined by measuring the average viscosities of four different commercial banana puddings on the market using an analog rotational viscometer (LT NDJ‐1, Nanjing, China), which were between 1540 and 2770 cP at 18°C. To compare and to be able to liken the consistency of puddings containing wheat flour with commercial puddings, banana pudding was chosen among the various types since it was assessed as the most compatible. For the determination of the pudding formulation in accordance with the targeted viscosity value, a sequential election was established. Firstly, experimental puddings with 3%, 5%, and 7% (w/w) of wheat flour were produced. The viscosities of the puddings were 620 cP, 1520 cP, and 3140 cP respectively, leading us to the second step with the 5% (w/w) wheat flour ratio (with a viscosity value almost at the targeted range) for the control puddings and the oleaster‐enriched puddings (gluten‐free counterparts) (Figure S1, Supporting Information). Then, 10 g of sugar, 5% (w/w) oleaster flours, and 1%, 1.5%, and 2% (w/w) sodium alginate, based on the milk weight, were added to 80 g of UHT milk. The resulting mixture was subjected to heat treatment at 85°C for 5 min, then cooled to room temperature and stored at 4°C for 24 h. Preliminary sensory evaluation conducted by the research team by comparing the appearances of the oleaster‐enriched experimental puddings with the commercial ones demonstrated that 1% (w/w) sodium alginate pudding resembles the commercial puddings in textural appearance (Figure S2, Supporting Information). Therefore, the favorable oleaster flour and sodium alginate ratios were found to be 5% (w/w) and 1% (w/w), respectively.

### 2.4 Preparation of Puddings and Sampling

For pudding production, 2 L of UHT milk was mixed with 5% wheat flour for C1, 5% wheat flour and 1% sodium alginate for C2, 5% oleaster flesh flour and 1% sodium alginate for OFP, and 5% oleaster peel flour and 1% sodium alginate for OPP (Table 1), with the help of a magnetic stirrer (Heidolph MR Hei‐Tec, Schwabach, Germany), and subjected to heat treatment at 85°C for 5 min in a shaking water bath (Heto SBD50, Harndrup, Denmark). The study was conducted using two independent production batches. The puddings were portioned in screw cap polypropylene cups (55 × 60 mm, D×H), cooled to room temperature, and stored at 4°C for 10 days. Analyses were carried out by sampling on the first and tenth days of storage. Wheat flour (WF) puddings without and with sodium alginate were regarded as controls and coded as C1 and C2, while OFF and OPF containing puddings were named as OF and OP, respectively.

**TABLE 1:** Design of the study.

Ingredients						
Sample	Milk (g)	Sugar (g)	Sodium alginate (g)	Wheat flour (g)	Oleaster flesh flour (g)	Oleaster peel flour (g)
C1	2000	250	—	100	—	—
C2	2000	250	20	100	—	—
OF	2000	250	20	—	100	—
OP	2000	250	20	—	—	100

*Note:* The amount of ingredients was calculated according to the total milk weight.

Abbreviations: C1, control sample 1; C2, control sample 2; OF, pudding sample with oleaster flesh flour; OP, pudding sample with oleaster peel flour.

### 2.5 Physicochemical Parameters

The moisture, ash, protein, and lipid contents of the flour samples were determined according to (AOAC 2000). The method of Marchetti et al. (2018) was used to determine the flour samples' water and oil absorption capacities. The UHT milk and pudding samples were subjected to analysis of titratable acidity, pH, dry matter, ash, milk fat, and total nitrogen (TN) (AOAC 2012). The protein content was calculated by multiplying the TN content with a conversion factor of 6.38 for UHT milk and pudding samples, 5.70 for wheat flour, and 6.25 for oleaster flours. The total dietary fiber content of the flour and pudding samples was analyzed with the Total Dietary Fiber Assay Kit (Megazyme, Wicklow, Ireland) by following the procedure described by Megazyme based on the method AOAC 985.29. The pudding samples were dried at 40°C and milled before analyzing for dietary fiber. For syneresis, 10 g of each pudding sample were transferred into tubes and centrifuged at 9000 × g for 15 min at 4°C (Nuve 800R, Türkiye). Syneresis was expressed as the percentage of drained water from the samples in Equation (1).

(1) $$\text{Syneresis}\;\left(\%\right)=\frac{\text{Weight of the water drained}\;\left(g\right)}{\text{Weight of the sample}\;\left(g\right)}\times 100$$

### 2.6 Phenolic Content and Antioxidant Activity

#### 2.6.1 Preparation of Extracts

The composition and the concentration of the extraction solution were determined with preliminary experiments by modifying the method of Zhang et al. (2022). Aqueous methanol was combined with the inorganic salt sodium citrate (50% methanol:2% (w/v) sodium citrate, 1:4 v/v). The samples (1 g) were mixed with 5 mL and 10 mL of extraction solution for the pudding and the flour samples, respectively. After the mixture was vortexed and then shaken (700 rpm) at room temperature (25°C ± 2°C) for an hour, it was centrifuged at 18,400 × g for 15 min at 4°C. The supernatants were passed through 0.45 μm of syringe filter (PTFE, Isolab, Germany) and maintained at −20°C. The extracts were used for the total phenolic content (TPC), phenolic composition, and antioxidant activity analyses.

#### 2.6.2 Determination of Phenolic Composition by Ultra High‐Performance Liquid Chromatography (UHPLC)

Phenolic composition of the flour and pudding samples was determined by a UHPLC (Thermo Scientific, Waltham, MA, USA) at Scientific, Industrial and Technological Application and Research Center of Abant İzzet Baysal University (Bolu, Türkiye). The method of (Mzoughi 2023) was modified as mobile phases of 88% ultra‐pure water −10% methanol (A) and 90% methanol—8% of ultra‐pure water (B), both containing 2% of acetic acid, with a gradient profile of 5% of B for 6.5 min, 5%–40% of B for 7.5 min, 40%–90% of B for 3 min, 90% of B for 3 min, and 5% of B for 1 min, with a flow rate of 0.4 mL/min. A C18 reversed‐phased column (4.6 × 250 mm, 5 μm) was used to identify and quantify the phenolic compounds (catechin, caffeic acid, ferulic acid, vanillic acid, gallic acid, rutin, chlorogenic acid, quercetin, and syringic acid), of which the standard curves were prepared at 250–6.25 μg/mL concentration before the sample run. The autosampler was set at 5 μL volume, the column temperature at 44°C, and the wavelength between 200 and 600 nm. The results were given in μg phenolic compound per g sample (μg PC/g sample).

#### 2.6.3 TPC Assay

TPC of flour and pudding samples was determined with Folin–Ciocalteu method according to the procedure of Ramírez‐Maganda et al. (2015). The extract or gallic acid (250 μL) was mixed with 1250 μL of 0.2 N Folin–Ciocalteu and vortexed. Then, 1000 μL of 7.5% sodium carbonate was added and re‐vortexed. The mixture was incubated at 50°C for 15 min in a water bath (Nuve ST30, Ankara, Türkiye), and the absorbance was measured at 750 nm (Shimadzu UV‐1800, Kyoto, Japan). Calibration curve (*y* = 12.652*x* + 0.0736, *R* ^2^ = 0.9995) of the gallic acid (10–60 mg/L) was used for the calculations and the results were expressed as μg gallic acid equivalent per g sample (μg GAE/g sample).

#### 2.6.4 2,2‐Diphenyl‐1‐Picrylhydrazyl (DPPH) Radical Scavenging Activity Assay

The antioxidant properties of flour and pudding samples were determined using the method of Hosseinipour et al. (2024) with minor modifications. The reaction mixture, 200 μL extract or Trolox and 2 mL of 75 μM DPPH, was incubated at room temperature (25°C ± 2°C) for 30 min in a dark place after vortexing. The absorbance was measured at 517 nm, and the radical scavenging activity was calculated according to Equation (2). The Trolox curve used for determining the antioxidant activity was generated by plotting the concentrations of Trolox (50–400 μM) against the radical scavenging activity (*y* = 0.1231*x* + 5.4325, *R* ^2^ = 0.9958). The results were given as μg Trolox equivalent per g sample (μg TE/g sample).

(2) $$\text{Radical scavenging activity}\;\left(\%\right)=\frac{A_{b}-A_{s}}{A_{b}}\times 100$$

where *A*_*b*_ is the absorbance of the blank, and *A*_*s*_ is the absorbance of the sample.

### 2.7 Microbiological Quality

In order to reveal the microbiological quality of pudding samples, total mesophilic aerobic bacteria count (TMAB) and yeast and mold count were performed (Anonymous 2005). Serial dilutions of the samples in ¼ Ringer's solution up to 10^−8^ were prepared. One mL of each dilution and 15 mL of Plate Count Agar (PCA) for TMAB and Potato Dextrose Agar (PDA) acidified with tartaric acid (10%) for yeast and mold count were inoculated by the pour plate method. After incubation for 24–48 h at 30°C–32°C for TMAB and 5 days at 25°C–28°C for yeast and mold, total viable counts in the samples were enumerated and calculated from Equation (3) by taking the weighted average of the results from two consecutive dilutions, considering petri dishes containing 25–250 colonies.

(3) $$N=\frac{C}{V\times \left(n_{1}+0.1\times n_{2}\right)\times d}$$

where *N* is the number of microorganisms in 1 g of the sample, *C* is the total number of colonies in all petri dishes counted, *V* is the volume (mL) transferred to the petri dishes counted, *n*_1_ is the number of petri dishes in which counting is made from the first dilution, *n*_2_ is the number of petri dishes in which counting is made from the second dilution, and *d* is the dilution ratio of the more concentrated of the two consecutive dilutions in which counting is made.

### 2.8 Color Analysis

The color (L*, a*, and b*) parameters according to the CIELab system were measured by a colorimeter (Konica Minolta CR 410, Sensing Inc., Osaka, Japan). Prior to analysis on storage days, the device was calibrated with standard white plate (*Y* = 85.90, *x* = 0.3168, *y* = 0.3242). Measurements were performed by filling the quartz container with a depth of approximately 1.5 cm and done twice for each sample. The total color difference was calculated based on control samples C1 and C2 as ∆*E*1* and ∆*E*2*, respectively, using Equation (4), where *L*_0_*, *a*_0_*, and *b*_0_* represent the color values of control samples whereas L*, a*, and b* stand for the color values of oleaster flour added puddings.

(4) $${\Delta E}^{*}=\sqrt{{\left(L^{*}-L_{0}^{*}\right)}^{2}+{\left(a^{*}-a_{0}^{*}\right)}^{2}+{\left(b^{*}-b_{0}^{*}\right)}^{2}}$$

### 2.9 Textural Parameters

Back extrusion test was conducted for the determination of textural parameters using a texture analyzer (TA‐XT, Stable Micro Systems, UK) equipped with a 5‐kg load cell and 35 mm back extrusion disc, according to Cartagena et al. (2024) with some modifications. The samples, each approximately 100 g, were analyzed in their original containers (55 × 60 mm, D×H) that were well‐suited with the back‐extrusion method according to the selected test conditions: pretest speed of 1 mm/s, test speed of 1 mm/s, posttest speed of 10 mm/s, distance of 20 mm, and trigger force of 5 g. Firmness (g), consistency (g.s), cohesiveness (g), and viscosity index (g.s) were the measured texture parameters.

### 2.10 Sensory Evaluation

Sensory evaluation was made using a 5‐point scale (ranging from 1‐disliked extremely to 5‐liked extremely), regarding the preferences of panelists about the color, appearance, texture, taste, and odor of the pudding samples. The evaluation group consisted of 44 semi‐trained panelists of both genders (22 female and 22 male), aged between 18 and 48, studying and working at Zonguldak Bülent Ecevit University (Zonguldak, Türkiye) and Ankara University (Ankara, Türkiye), who consume pudding regularly. Before the evaluation, panelists were informed about the puddings' ingredients, informed consent was obtained from each panelist, and oral instructions were given on how to conduct the test. Puddings were coded with three‐digit random numbers to avoid expectancy effects. Participants received 20 g of each pudding sample in plastic cups in a fully randomized order, and tasteless‐odorless crackers and water were also offered to cleanse their palate between samples to prevent the carry‐over effect. The sensory evaluation was approved by the Human Research Ethics Committee of Zonguldak Bülent Ecevit University on 05.09.2024, with Protocol Number 761. The research ethics committee approval of the study could be presented in case of any demand.

### 2.11 Statistical Analysis

The study was conducted using two independent production batches and analytical measurements for each batch were performed in duplicate. Statistical analyses were performed by SPSS Statistics (v.22.0). The differences between the storage days of the same samples were compared by the one‐way analysis of variance (ANOVA) (*p* < 0.05) followed by Tukey's/Games‐Howell post hoc tests, and the differences between the samples at the same storage days were analyzed by independent samples *t*‐test. Data were expressed as mean values ± standard deviation when indicated.

## 3 Results and Discussion

### 3.1 Characterization of Milk and Flour Samples

The physicochemical properties of the UHT milk were determined as follows: 6.73 ± 0.04 pH, 6.88% ± 0.18% titratable acidity as lactic acid, 11.29% ± 0.05% dry matter, 0.68% ± 0.01% ash, 2.68% ± 0.00% protein, and 3.00% ± 0.00% milk fat. Proximate composition of flours is given in Table 2. There were statistically significant differences between the physicochemical properties of WF, OFF, and OPF (*p* < 0.05). The protein and lipid contents of WF were almost twice as much as those of oleaster flours. On the other hand, dry matter, ash, water, and oil absorption capacities of oleaster flours were greater than those of WF. The dietary fiber content, TPC and antioxidant activity of oleaster flours were noticeably much higher than those of WF. The proximate composition of the oleaster flours was generally similar to the findings of previous studies (Sahan et al. 2015; Şahin 2023a, 2023b, 2024). Flours with higher water absorption capacity (WAC) can be expedient components of foods such as batters, soups, and gravies, which require viscosity to some extent. On the other hand, a higher oil absorption capacity (OAC) makes flour a favorable ingredient for improved mouthfeel and flavor retention (Jan et al. 2022). The higher WAC of oleaster flour compared to WF may be attributed to its elevated dietary fiber content (Sahan et al. 2015). However, there may be other hydrophilic components in oleaster flesh, such as soluble carbohydrates and possible pectinic compounds that can form a viscous solution (Huojiaaihemaiti et al. 2022), as both OFF and OPF had similar WAC, even though the OPF contained significantly more dietary fiber than OFF (*p* < 0.05). Furthermore, the insoluble dietary fiber may be higher in the peel, which may lead to similar WAC values with flesh. The WAC and OAC values of oleaster flours showed that by binding both hydrophilic and hydrophobic components, they could be potential ingredients of pudding‐type dairy products, which could also be functional thanks to the dietary fiber content, phenolic compound content, and antioxidant activity of oleaster flours. OFF and OPF involved 4.9‐fold and 6.8‐fold higher dietary fiber, and 4.2‐fold and 4.5‐fold higher total phenolic compounds, respectively, compared to WF (*p* < 0.05). While the antioxidant activity of wheat flour could not be determined under the analysis conditions, OFF and OPF were found to have 1838.55 μg TE/g sample and 1738.82 μg TE/g sample antioxidant activity, respectively (*p* < 0.05).

**TABLE 2:** Proximate composition of flours.

Physicochemical properties	WF	OFF	OPF
Dry matter (%)	92.67 ± 0.14^c^	95.93 ± 0.03^b^	96.82 ± 0.16^a^
Ash (%)	0.46 ± 0.04^c^	2.20 ± 0.08^a^	2.03 ± 0.10^b^
Protein (%)	11.54 ± 0.04^a^	6.50 ± 0.09^b^	6.12 ± 0.11^b^
Lipid (%)	1.22 ± 0.06^a^	0.52 ± 0.06^b^	0.82 ± 0.04^b^
Water absorption capacity (g/g sample)	0.70 ± 0.02^b^	2.19 ± 0.25^a^	2.01 ± 0.07^a^
Oil absorption capacity (g/g sample)	0.76 ± 0.01^c^	1.94 ± 0.02^a^	1.38 ± 0.01^b^
Dietary fiber (%)	4.85 ± 0.16^c^	23.69 ± 0.26^b^	32.85 ± 0.58^a^
Total phenolic compound (μg GAE/g sample)	436.07 ± 97.39^b^	1844.25 ± 191.89^a^	1959.95 ± 96.29^a^
Antioxidant activity (μg TE/g sample)	nd	1838.55 ± 24.36^a^	1738.82 ± 103.93^a^

*Note:* Different lower‐case letters in the same line indicate significant differences between samples (*p* < 0.05).

Abbreviations: OFF, oleaster flesh flour; OPF, oleaster peel flour; WF, wheat flour.

### 3.2 Physicochemical Properties of Pudding Samples

The changes in physicochemical properties of pudding samples during storage are given in Table 3. There were no statistically significant differences in the physicochemical properties of the samples between the storage days, except for the pH value of OF and OP. The pH value of these samples was higher on the tenth day. A slight increase in the pH of soy‐based desserts during 60‐day storage was also observed by Granato et al. (2010). Leal et al. (2022) related the pH increase of their creamy dairy dessert with white chocolate flavor during storage to the proteins that might show buffering activity and action. Citric and malic acids were reported to be the dominant organic acids in oleaster (Sahan et al. 2015). Therefore, the titratable acidity is shown in Table 3 in terms of different organic acid equivalents. The titratable acidity values were higher in OF and OP samples compared to control samples, which is believed to be linked to oleaster flour enrichment (Çalışkan 2021). Similarly, Popova et al. (2024) found the titratable acidity of puddings with apricot and plum powder to be higher than that of the control sample in terms of citric acid equivalent.

**TABLE 3:** Physicochemical properties of pudding samples during storage.

Physicochemical properties	Storage period (days)	
	1	10
pH		
C1	6.34 ± 0.14^aB^	6.41 ± 0.04^aC^
C2	6.66 ± 0.11^aA^	6.82 ± 0.02^aA^
OF	6.56 ± 0.05^bA^	6.68 ± 0.04^aB^
OP	6.52 ± 0.02^bA^	6.63 ± 0.03^aB^
Titratable acidity (l.a. %)		
C1	0.15 ± 0.01^aB^	0.15 ± 0.01^aB^
C2	0.12 ± 0.00^aB^	0.12 ± 0.00^aB^
OF	0.30 ± 0.07^aA^	0.24 ± 0.05^aA^
OP	0.28 ± 0.03^aA^	0.24 ± 0.02^aA^
Titratable acidity (m.a. %)		
C1	0.23 ± 0.02^aB^	0.23 ± 0.02^aB^
C2	0.18 ± 0.00^aB^	0.18 ± 0.00^aB^
OF	0.44 ± 0.10^aA^	0.36 ± 0.07^aA^
OP	0.42 ± 0.04^aA^	0.36 ± 0.03^aA^
Syneresis (%)		
C1	30.28 ± 3.98^aA^	45.07 ± 23.03^aA^
C2	0^aB^	0^aB^
OF	0.10 ± 0.14^aB^	5.94 ± 6.33^aB^
OP	0.24 ± 0.41^aB^	3.18 ± 4.71^aB^
Dry matter (%)		
C1	28.56 ± 1.85^aA^	28.81 ± 1.27^aA^
C2	25.78 ± 0.19^aB^	26.68 ± 0.18^aB^
OF	28.76 ± 1.96^aA^	28.59 ± 1.65^aA^
OP	28.95 ± 1.07^aA^	28.74 ± 0.59^aA^
Ash (%)		
C1	0.69 ± 0.03^aB^	0.66 ± 0.03^aC^
C2	0.73 ± 0.03^aB^	0.73 ± 0.01^aB^
OF	0.99 ± 0.14^aA^	0.97 ± 0.07^aA^
OP	0.96 ± 0.01^aA^	0.94 ± 0.01^aA^
Protein (%)		
C1	3.96 ± 0.25^aA^	3.96 ± 0.23^aA^
C2	3.78 ± 0.14^aA^	3.65 ± 0.06^aA^
OF	3.59 ± 0.06^aA^	3.75 ± 0.28^aA^
OP	3.56 ± 0.11^aB^	3.54 ± 0.06^aB^
Dietary fiber (%)		
C1	1.13 ± 0.08^aC^	0.92 ± 0.20^aC^
C2	1.82 ± 0.07^aB^	1.76 ± 0.12^aB^
OF	3.28 ± 0.45^aA^	2.89 ± 0.30^aA^
OP	3.11 ± 0.17^aA^	3.02 ± 0.06^aA^

*Note:* The lower‐case letters in the same line indicate that the differences between the storage days of the same pudding sample are significant (*p* < 0.05). The upper‐case letters in the same column indicate that the differences between the pudding samples on the same storage day are significant (*p* < 0.05).

Abbreviations: C1, control sample 1; C2, control sample 2; l.a., lactic acid; m.a., malic acid; OF, pudding sample with oleaster flesh flour; OP, pudding sample with oleaster peel flour.

Syneresis is defined as the free water leached out from a solid under centrifugal forces (Moin et al. 2017). It is reported that using sodium alginate in foods can reduce syneresis (Qin et al. 2018). The low values of syneresis in OF and OP, particularly in C2, were clearly induced by the addition of sodium alginate. Sodium alginate is heat‐stable and known to form stable gels in the presence of Ca^+2^ and casein, which makes it an important ingredient in UHT‐processed puddings (Chandan and Kilara 2016). Because syneresis impacts appearance, obtaining lower syneresis values is crucial for the product's acceptability (Celeghin et al. 2016). Although the increase was not statistically significant, higher syneresis was observed on the tenth day. Since oleaster flour is known to include a low amount of starch in the range of 0.013%–0.043%, depending on the genotype and the part of the fruit (Sahan et al. 2015), reorganization and retrogradation of the starch during cold storage may be the reason for the syneresis increase in the OF and OP (Rodriguez Furlán and Campderrós 2017). Despite the low amount of starch, because the flesh can contain more starch than the peel, the alteration of the syneresis of OF was much more prominent. Furthermore, structural changes, such as fiber–fiber interaction within the gel network, could affect syneresis during storage (Chen et al. 2025).

The lowest dry matter content among the samples was observed for C2 (*p* < 0.05). Probably due to the water retention capacity of the sodium alginate, C2 had higher moisture content than C1, which lacks sodium alginate. Both sodium alginate and oleaster fibers can bind water. Sodium alginate is a water‐soluble polysaccharide (Shen and Hsieh 2014), and it was also found by Hou and Wu (2019) that, owing to the strong hydrophilicity, sodium alginate film could contain amounts of water even after 24 h of vacuum drying. On the other hand, oleaster contains both soluble and insoluble dietary fibers and also the insoluble fiber ratio is higher (Şahin 2023a, 2023b). Furthermore, it is reported that due to the porous surface, water may be retained by insoluble dietary fibers physically (Wang et al. 2021) and soluble fibers have more active groups and better water absorption than insoluble fibers (Wang et al. 2024). Moreover, while sodium alginate generates heat‐stable gels (Chandan and Kilara 2016), it was determined that the water absorption of oleaster polysaccharides has decreased with increasing temperatures, and at low temperatures, insoluble fibers are capable of retaining much water (Sharifian‐Nejad and Shekarchizadeh 2019). Therefore, probably at the beginning of ingredient mixing of puddings, insoluble fibers in OF and OP competed for water with the sodium alginate. However, insoluble fibers might have lost some of the entrapped water during heat processing.

The differences in ash contents of the samples were thought to be related to the high mineral content of oleaster flours (Table 2). It was indicated that oleaster exocarp, endocarp (Şahin 2023a), and mesocarp flours (Şahin 2023b) had high ash and ample mineral content. The protein content of the OP was statistically lower than that of the other samples (*p* < 0.05) (Table 3). This was consistent with the protein contents of the flours (Table 2).

Since sodium alginate is an indigestible polysaccharide and can be considered a form of dietary fiber (Brownlee et al. 2005), the dietary fiber content of C2 was found to be higher than that of C1 (*p* < 0.05). Meanwhile, OF and OP had nearly twice as much dietary fiber content of as the control samples (*p* < 0.05), which is one of the important outputs of this study. Oleaster flour is a rich source of dietary fiber compared the WF (Table 2), and therefore, using oleaster flour resulted in elevated dietary fiber content in pudding samples of OF and OP. According to the recommendation of the Food and Drug Administration (FDA), based on a 2000‐cal daily diet, the daily value of dietary fiber should be at least 28 g per day. Furthermore, it is also suggested that when choosing foods, the daily value per serving should be taken into consideration, and this value should be in the range of 5%–20% (FDA 2021). Therefore, when considering the mean weight of commercial puddings as 70 g, the daily value for dietary fiber of OP and OF corresponded to 7.8% and 8.2%, respectively, which is such a good achievement for a dessert‐type product.

### 3.3 TPC of Pudding Samples

**FIGURE 1:**
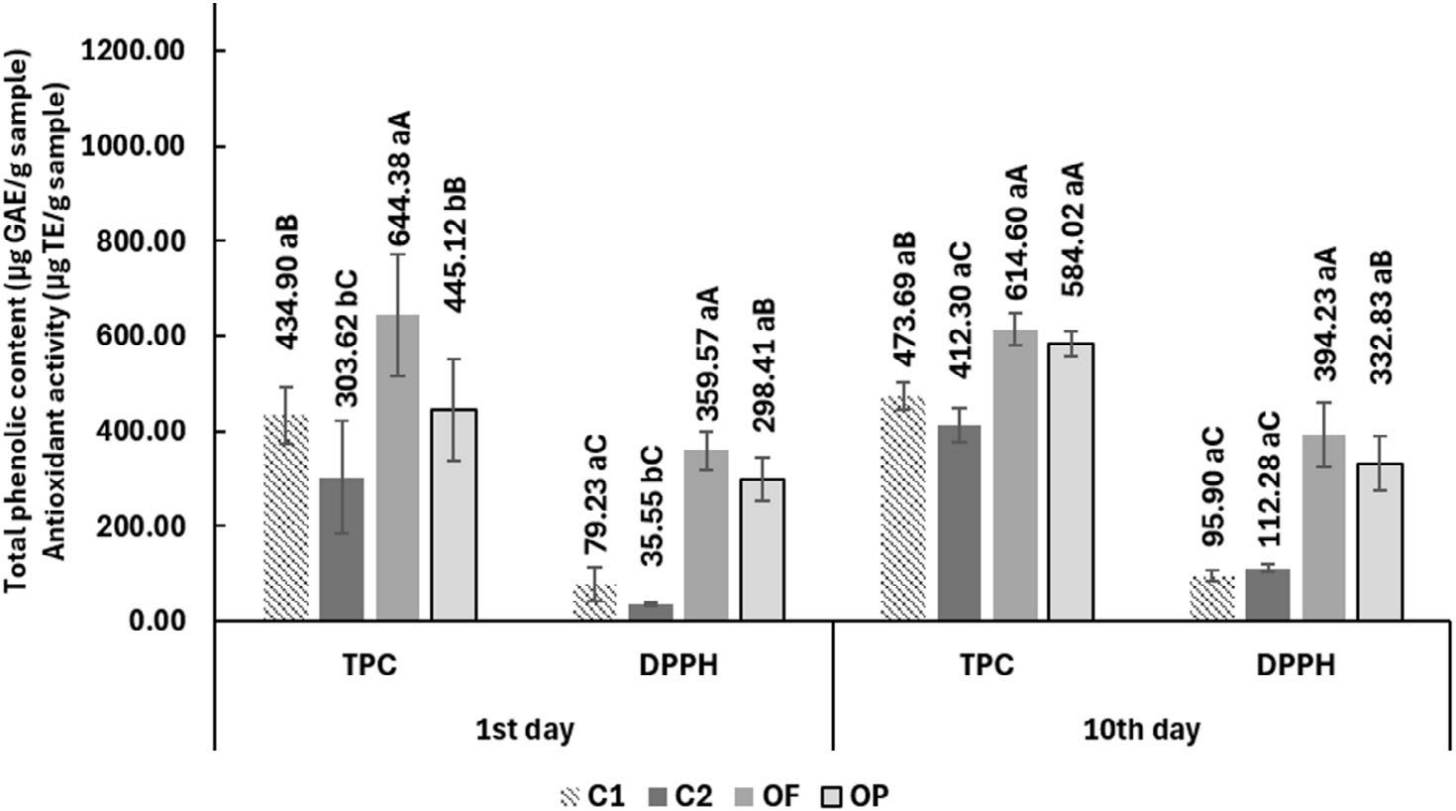
Total phenolic content and antioxidant activity of the pudding samples during storage. The lowercase letters indicate that the differences between the storage days of the same pudding sample are significant (*p* < 0.05). The uppercase letters indicate that the differences between the pudding samples on the same storage day are significant (*p* < 0.05). C1, control sample 1; C2, control sample 2; OF, pudding sample with oleaster flesh flour; OP, pudding sample with oleaster peel flour. Data are given on fresh weight.

The TPC of pudding samples and its value at the end of the storage are shown in Figure 1. Among the pudding samples, while OF and C2 had the highest (644.38 μg GAE/g sample) and the lowest (303.62 μg GAE/g sample) TPC (*p* < 0.05) on the first storage day, respectively, no statistical difference was observed between the TPC of C1 (434.90 μg GAE/g sample) and OP (445.12 μg GAE/g sample). Given that sodium alginate is used as an encapsulation agent for bioactive substances (Qin et al. 2018), in cases of phenolic substance entrapment in puddings by sodium alginate, phenolic compounds were extracted with the help of sodium citrate during analyses to disrupt the capsule. Therefore, the lower TPC of C2 compared to C1 may be either due to the dilution effect caused by the sodium alginate addition, or sodium citrate may not have been sufficient to release all phenolics encapsulated by sodium alginate due to the gluten‐sodium alginate interaction. As expected, OF showed significant differences compared to C1 and C2 in terms of TPC (*p* < 0.05). Although the differences in TPC between OFF and OPF were statistically insignificant (Table 2), OF exhibited a higher TPC than OP. This may be related to the ratio of free and bound phenolics in different parts of the oleaster. It was reported that phenolic compounds can be covalently bound to indigestible components, specifically dietary fiber (Du et al. 2022). Additionally, the bound phenolic compounds in plants are typically found in protective tissues such as the pericarp (Rocchetti et al. 2022). Furthermore, phenolic compounds are known to interact covalently or non‐covalently with milk proteins, resulting in either irreversible or reversible binding. And milk proteins are regarded as potential transport systems for bioactive compounds (Han et al. 2019; Yildirim‐Elikoglu and Erdem 2018). Probably phenolics in the OP were more prone to interact with other molecules in the pudding matrix, which led to a decrease in its TPC. It is also determined that there was a statistically significant increase in the TPC value of C2 and OP after 10 days of cold storage (*p* < 0.05). Considering the raw values, TPC of all puddings, except OF, increased after 10 days of storage. It is thought that sodium alginate may have served as a protective agent for the phenolic compounds during storage. The reason for the increase may also be the reversible interactions of phenolics that slow down their release. During storage, some of the reversibly bound phenolics could have been released. García‐Alonso et al. (2003) stored the desserts containing grape, blackberry, blackcurrant, raspberry, and cherry concentrates for 12 months at three different temperatures (8°C, 21°C, and 30°C) and found that even at the lowest temperature, anthocyanins, hydroxycinnamic acids, flavonols, and stilbenoids decreased while ellagic acids tended to increase based on their release from ellagitannins. Even though the TPC of oleaster enriched puddings were higher than fig‐milk desserts (294–377 μg GAE/g) (Zare et al. 2024), it is well predicted that the TPC of the enriched products depends on the type and concentration of the fortifying material.

### 3.4 Phenolic Composition of Flours and Pudding Samples

Table 4 shows the phenolic composition of flours and pudding samples. Considering the flours, gallic acid and protocatechuic acid content of WF was approximately 2‐fold higher than those of OFF and OPF (*p* < 0.05). On the other hand, catechin, ferulic acid, rutin, and phloridzin were not determined in WF. While all flour samples had statistically similar caffeic acid content, quercetin was not determined in OFF and OPF. OPF had higher chlorogenic acid, vanillic acid, and ferulic acid, and OFF had higher syringic acid and phloridzin (*p* < 0.05). Remarkably, rutin was found only in OPF, and phloridzin in OFF. Results indicated that catechin is the primary phenolic component of both OFF and OPF. Catechin was followed by ferulic acid, chlorogenic acid, rutin, and gallic acid in OPF, along with chlorogenic and gallic acids in OFF. Karkar and Şahin (2022) found the main phenolics in different parts of oleaster as gallic acid, catechin, and their derivatives. Contrary to our findings, Ayaz and Bertoft (2001) determined the most abundant phenolic components in oleaster fruit as caffeic acid and 4‐hydroxybenzoic acid, while Şahin and Aybastıer (2024) found that rutin content was the highest and followed by protocatechuic, caffeic, and ferulic acid, respectively, in oleaster extract. Also, ferulic acid, vanillic acid, 4‐hydroxybenzoic acid (Dilek Tepe and Doyuk 2020), chlorogenic acid and caffeic acid (Carradori et al. 2020) were found as leading phenolic compounds in oleaster flours and fruits, indicating a season or species‐dependent difference.

**TABLE 4:** Phenolic compound contents of flour and pudding samples (μg/g sample).

Phenolic compound	Wheat flour	Oleaster flesh flour	Oleaster peel flour	C1		C2		OF		OP	
				Storage period (days)		Storage period (days)		Storage period (days)		Storage period (days)	
				1	10	1	10	1	10	1	10
Gallic acid	53.89 ± 2.84^a^	27.07 ± 1.26^b^	22.46 ± 1.70^b^	nd	nd	2.41 ± 0.18	nd	nd	nd	nd	nd
Protocatechuic acid	4.48 ± 0.01^a^	1.96 ± 0.13^b^	2.48 ± 0.09^b^	0.17 ± 0.04^a^	0.09 ± 0.04^b^	0.10 ± 0.00^a^	0.01 ± 0.00^a^	2.08 ± 0.06^b^	2.98 ± 00^a^	2.37 ± 0.32^a^	2.46 ± 0.00^a^
Chlorogenic acid	40.80 ± 0.12^b^	29.10 ± 1.10^c^	67.19 ± 4.52^a^	0.05 ± 0.01^a^	0.03 ± 0.00^a^	nd	nd	1.42 ± 0.13^a^	1.11 ± 0.04^a^	2.42 ± 0.06^a^	2.14 ± 0.05^b^
Caffeic acid	0.56 ± 0.21^a^	0.21 ± 0.06^a^	0.45 ± 0.17^a^	0.31 ± 0.05^a^	0.32 ± 0.00^a^	0.26 ± 0.03^a^	0.36 ± 0.00^a^	0.33 ± 0.00^b^	0.35 ± 0.00^a^	0.16 ± 0.01^b^	0.30 ± 0.00^a^
Vanillic acid	2.11 ± 0.01^b^	2.53 ± 0.06^b^	4.18 ± 0.03^a^	0.03 ± 0.03^a^	0.02 ± 0.01^a^	0.06 ± 0.05^a^	0.15 ± 0.00^a^	0.16 ± 0.00^a^	0.12 ± 0.07^a^	0.23 ± 0.04^a^	0.20 ± 0.00^a^
Quercetin	18.90 ± 0.05	nd	nd	nd	nd	nd	nd	nd	nd	nd	nd
Syringic acid	0.42 ± 0.00^b^	3.26 ± 0.01^a^	0.88 ± 0.00^b^	0.14 ± 0.06^a^	0.11 ± 0.00^a^	nd	nd	nd	nd	nd	0.04 ± 0.00
Catechin	nd	134.98 ± 0.28^a^	79.73 ± 5.20^b^	nd	nd	nd	nd	nd	nd	nd	1.92 ± 0.54
Ferulic acid	nd	4.23 ± 0.29^b^	75.88 ± 6.03^a^	nd	nd	nd	nd	nd	nd	nd	nd
Rutin	nd	nd	26.20 ± 3.06	nd	nd	nd	nd	nd	nd	nd	nd
Phloridzin	nd	2.44 ± 0.01	nd	nd	nd	nd	nd	nd	nd	nd	nd

*Note:* Flour samples; the lower cases in the same line (a, b) indicate that the differences between the flour samples are significant (*p* < 0.05). Pudding samples; C1, control sample 1; C2, control sample 2; OF, pudding sample with oleaster flesh flour; OP, pudding sample with oleaster peel flour; nd, non‐defined. The lower‐case letters in the same line indicate that the differences between the storage days of the same pudding sample are significant (*p* < 0.05). Statistical evaluations were made for flour and pudding samples separately. Data for pudding samples are given on fresh weight.

Abbreviation: nd, non‐defined.

The difference in phenolic composition of puddings between the storage days was statistically significant in protocatechuic acid content of C1 and OF, caffeic acid content of OF, and chlorogenic and caffeic acid contents of OP (*p* < 0.05, Table 4). Furthermore, although syringic acid and catechin were not detected in OP on the first day of storage, they were identified after 10 days of cold storage, probably due to their slow‐release rate from the encapsulation agent, sodium alginate. Even though gallic acid was identified in all flour samples, particularly at twice the level in WF, it was not detected in pudding samples, except for C2 on day 1, in a very small amount. Similarly, the chlorogenic, vanillic, ferulic acids, catechin, rutin, and phloridzin contents of puddings were either much lower compared to the flours or not detected. Although quercetin was found in WF in high amounts, it was not detected in any of the control puddings, like syringic acid in puddings with OFF. These might be explained by the destructive effect of heat treatment on the phenolic compounds during pudding preparation. On the other hand, the dilution effect should be considered since the puddings contained 5% of flour. Moreover, their specific interaction with alginate may be different, which could affect their release during extraction procedure. The protocatechuic acid content of control puddings was so low; on the contrary, it was higher in oleaster puddings despite the low content of oleaster used in the formula. This may also be related to the phenolic content of milk (Ertan et al. 2017; Vázquez et al. 2015).

### 3.5 Antioxidant Activity of the Pudding Samples

The DPPH radical scavenging activities of pudding samples are given in Figure 1. In terms of antioxidant activities, the order of the samples from the highest to the lowest was OF > OP > C1 > C2, as it was in the TPC. The differences among the samples were significant (*p* < 0.05), except C1 and C2. There were no differences in the antioxidant activity of C1, OF, and OP when comparing the storage days. On the contrary, the increase in the antioxidant activity of C2 on the tenth day was statistically significant (*p* < 0.05). As mentioned in TPC, this may be due to the release of some of the phenolic compounds during storage. The TPC and antioxidant activity of the oleaster enriched puddings were higher compared to controls as expected, based on the phenolic content of oleaster fruits. Rather than OPF, the effect of OFF was substantially (*p* < 0.05) higher in increasing the antioxidant potential of the puddings. Although no study on oleaster‐enriched puddings was found in literature for comparison, saffron‐enriched pudding formulations had lower antioxidant activity (Chobdar Rahim 2017) compared to the puddings in our study. It was also determined in various studies that oleaster was effective in increasing the antioxidant activity of different products, such as cookies (Sahan et al. 2019; Şahin 2023a), crackers (Düşkün et al. 2025; Incedayi and Erol 2023), tarhana (Şahin et al. 2024), and kefir (Gül et al. 2023).

### 3.6 Microbiological Quality of the Pudding Samples

Table 5 shows the microbiological counts of the pudding samples during storage. No yeast and mold growth was observed during the storage of the samples, and the storage did not affect the TMAB counts statistically. The TMAB counts of the samples ranged from 1.26 to 2.14 log cfu/g, and OF and OP had nearly twice the levels of the control samples. This was probably due to the initial microbial load of the flour samples. Furthermore, the higher sugar content of oleaster (Ayaz and Bertoft 2001) compared to WF might have promoted bacterial growth. Similarly, (Seyrekoğlu et al. 2025) observed higher mesophilic microbial counts in sugar‐containing rice puddings. It is obvious from the TMAB, yeast, and mold count of the controls and oleaster‐enriched puddings that they were microbiologically safe to consume, according to Microbiological Requirements of Food Groups (FAO 2000) with safety acceptance limits of 4 log cfu/g for TMAB and 3 log cfu/g for yeast counts.

**TABLE 5:** Microbiological counts of pudding samples during storage.

Microbiological counts (logcfu/g)	Storage period (days)	
	1	10
TMAB		
C1	1.26 ± 0.36^aB^	1.65 ± 0.00^aB^
C2	1.33 ± 0.46^aB^	1.41 ± 0.34^aB^
OF	2.01 ± 0.38^aA^	2.13 ± 0.45^aA^
OP	2.45 ± 0.18^aA^	2.14 ± 0.24^aA^
Yeast and mold		
C1	nd	nd
C2	nd	nd
OF	nd	nd
OP	nd	nd

*Note:* The lower‐case letters in the same line indicate that the differences between the storage days of the same pudding sample are significant (*p* < 0.05). The upper‐case letters in the same column indicate that the differences between the pudding samples on the same storage day are significant (*p* < 0.05).

Abbreviations: C1, control sample 1; C2, control sample 2; cfu, colony forming unit; nd, non‐defined; OF, pudding sample with oleaster flesh flour; OP, pudding sample with oleaster peel flour; TMAB, total mesophilic aerobic bacteria.

### 3.7 Color Evaluation of the Pudding Samples

The color parameters of the pudding samples are shown in Table 6. There were statistically significant differences in the color parameters in almost all the samples during storage (*p* < 0.05). A significant decrease (*p* < 0.05) was observed in the puddings' L* and b* values, except for C2, on the tenth day of storage. For a* value, the increase was only significant for C1 and OP (*p* < 0.05). The variations in the L*, a*, and b* values showed that puddings became darker, redder, and less yellow during storage. One of the reactions responsible for the changes in puddings during storage is reported to be the Maillard reaction, nonenzymatic browning (Fan et al. 2023; Moufle et al. 2018). A slight browning may explain the changes in the L*, a*, and b* values of the puddings shown in Table 6 during storage. L* and a* values of the control samples on the same storage day were higher than the oleaster flour‐enriched samples (*p* < 0.05). On the other hand, the highest and the lowest b* values were observed for C2 and OP, respectively (*p* < 0.05). Image of flour and pudding samples are shown in Figure S3 (Supporting Information). The total color difference (∆E) indicates the degree of difference from the reference and scaled as the difference is very distinct (∆E* > 3), distinct (1.5 < ∆E* < 3) and small difference (∆E* < 1.5) (Pathare et al. 2013). The use of sodium alginate in the pudding recipe created distinct differences on the first day (2.49 ± 2.06) and on the tenth day (3.53 ± 0.91) among the control samples. The total color difference values, ∆*E*1* and ∆*E*2*, showed that use of both OFF and OPF caused a significant change in color of the puddings (*p* < 0.05). Furthermore, the effect of OPF on the color was much more apparent. ∆*E*1* of OF and OP was significantly higher (*p* < 0.05) on the tenth day, while no significant difference was observed for ∆*E*2* (Table 6).

**TABLE 6:** Color and textural parameters of pudding samples during storage.

Parameters	Storage period (days)	
	1	10
L*		
C1	72.66 ± 0.38^aA^	66.49 ± 0.35^bA^
C2	70.33 ± 0.21^aB^	62.99 ± 1.27^aB^
OF	60.31 ± 1.43^aC^	52.59 ± 1.56^bC^
OP	50.20 ± 1.20^aD^	42.45 ± 1.06^bD^
a*		
C1	−1.66 ± 0.17^aC^	−1.52 ± 0.16^bC^
C2	−1.81 ± 0.28^aC^	−1.53 ± 0.39^aC^
OF	−3.72 ± 0.54^aB^	−3.30 ± 0.39^aB^
OP	−6.51 ± 0.12^aA^	−5.29 ± 0.13^bA^
b*		
C1	10.61 ± 0.09^aB^	9.09 ± 0.04^bA^
C2	11.45 ± 0.08^aA^	9.14 ± 0.58^aA^
OF	10.96 ± 0.13^aB^	8.56 ± 0.01^bA^
OP	8.58 ± 0.45^aC^	6.11 ± 0.40^bB^
∆*E*1*		
C2	2.49 ± 0.23^aC^	3.53 ± 0.91^aC^
OF	13.48 ± 1.95^bB^	14.73 ± 1.99^aB^
OP	23.99 ± 1.53^bA^	25.17 ± 1.41^aA^
∆*E*2*		
C1	2.49 ± 0.23^aC^	3.53 ± 0.91^aC^
OF	11.45 ± 1.82^aB^	11.49 ± 2.92^aB^
OP	21.96 ± 1.41^aA^	21.86 ± 2.41^aA^
Firmness (g)		
C1	26.32 ± 2.06^aB^	34.55 ± 7.96^aB^
C2	151.42 ± 74.06^aA^	140.13 ± 42.75^aA^
OF	155.75 ± 64.63^aA^	225.49 ± 136.35^aA^
OP	140.34 ± 5.98^aA^	171.94 ± 17.89^aA^
Consistency (g.s)		
C1	447.94 ± 23.28^aB^	508.29 ± 86.01^aB^
C2	2429.43 ± 1121.52^aA^	2232.67 ± 705.06^aA^
OF	2535.49 ± 1122.62^aA^	3469.92 ± 2053.11^aA^
OP	2276.01 ± 118.53^bA^	2644.59 ± 131.30^aA^
Cohesiveness (g)		
C1	20.03 ± 2.52^aB^	20.25 ± 0.10^aB^
C2	94.06 ± 6.18^aA^	87.19 ± 12.84^aA^
OF	92.11 ± 1.61^aA^	100.04 ± 12.88^aA^
OP	95.08 ± 2.85^aA^	96.47 ± 4.09^aA^
Index of viscosity (g.s)		
C1	27.03 ± 9.91^aC^	23.45 ± 6.00^aC^
C2	124.44 ± 64.09^aB^	153.92 ± 0.29^aB^
OF	164.32 ± 28.89^aA^	182.24 ± 3.15^aA^
OP	199.96 ± 27.76^aA^	183.20 ± 6.58^aA^

*Note:* Cohesiveness and index of viscosity values are given in absolute values. The lower‐case letters in the same line (a, b) indicate that the differences between the storage days of the same pudding sample are significant (*p* < 0.05). The upper‐case letters in the same column (A–D) indicate that the differences between the pudding samples on the same storage day are significant (*p* < 0.05).

Abbreviations: ∆*E*1*, total color difference calculated according to C1; ∆*E*2*, total color difference calculated according to C2; a*, ranging from red (+a*) to green (−a*); b*, yellow (+b*) to blue (−b*); C1, control sample 1; C2, control sample 2; L*, luminosity ranging from 0 (black) to 100 (white); OF, pudding sample with oleaster flesh flour; OP, pudding sample with oleaster peel flour.

### 3.8 Textural Evaluation of the Pudding Samples

The textural parameters, which were evaluated by firmness, consistency, cohesiveness, and viscosity index values, are given in Table 6. There were no statistically significant differences in the textural parameters of pudding samples (except for OP in consistency) during 10 days of storage. Firmness values of all samples increased as raw values during the storage period but were not found to be significant, in contrast to (Fan et al. 2019) who observed a significant increase in the firmness of puddings involving fish gelatin, sodium alginate, and virgin coconut oil after 3 weeks. The textural parameters of the samples were lower than whey‐based desserts containing carrageenan and carob powder (Şanlı 2023). Considering the same storage day, significant differences between C1 and other samples were observed for textural parameters (*p* < 0.05). C2, OF, and OP got higher firmness values, so thicker texture than C1 due to compositional differences. When compared to C1, other samples differ in sodium alginate content. Additionally, OF and OP were rich in soluble and insoluble dietary fibers that impart water absorption. Consistency defines the degree of thickness of the sample (Ciron et al. 2010). In back extrusion analysis, cohesiveness can be defined as the force necessary to withdraw the probe from the product (Lima and Guraya 2005), and the higher the viscosity index means the more resistance to withdrawing from the sample (Syahariza and Yong 2017). C1 was significantly (*p* < 0.05) lower than the other samples on both storage days in terms of consistency, cohesiveness, and viscosity index. The highest values for consistency and cohesiveness were observed for OF both on the first and the tenth day. A significant increase in consistency was observed in OP during storage (*p* < 0.05). In terms of viscosity index, OF and OP had the highest value (*p* < 0.05), and this may be attributed to the higher dietary fiber content of oleaster flours (Table 2), which can compete for water. The viscosity index values clearly indicate that OFF and OPF, along with sodium alginate, significantly enhanced the texture of the pudding samples, thereby increasing their potential for commercialization.

### 3.9 Sensory Evaluation of Pudding Samples

**FIGURE 2:**
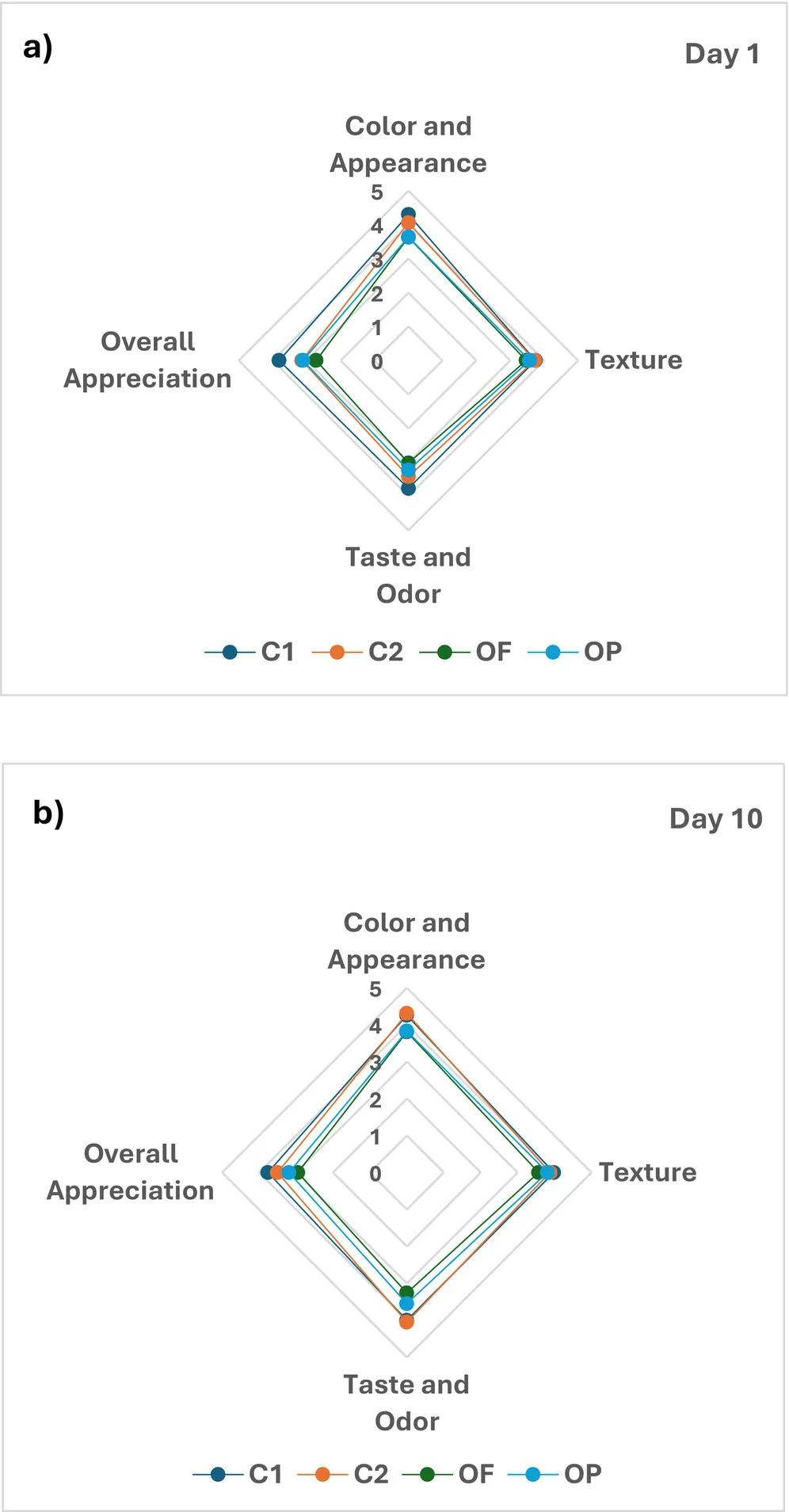
Graphical representation of the sensory evaluation results of puddings: (a) Day 1, (b) Day 10.

The graphical representation of the sensory evaluation of the puddings is seen in Figure 2. There were no statistically significant differences in sensory scores of pudding samples between the storage days, except for the taste and odor of C2 and OP, and overall appreciation of C2 (*p* < 0.05). No significant differences were observed among the textures of the samples on the same storage days. In terms of color and appearance, OF and OP had lower scores than C1 and C2 on day 1 (*p* < 0.05). However, on the tenth day, the color and appearance scores of all samples were statistically similar. Since color and appearance parameters are significant for the initial quality and acceptability of the fruit‐based dairy products (Zare et al. 2024), it is concluded that oleaster‐enriched puddings were as much appreciated as control samples. Furthermore, the taste and odor of OF on day 1 and OF and OP on day 10 were less liked (*p* < 0.05), and the overall appreciation of C1 on day 1 and C1, OF, and OP on day 10 was preferred among all the pudding samples (*p* < 0.05). Generally, the control pudding C1 scored slightly higher than C2; however, some panelists complained about the syneresis and watery texture in C1, while others highlighted the unpleasantly thick and lumpy texture of C2. Although the overall appreciation score of C1 was the highest, among the oleaster‐containing samples, OP had close scores to C1, and after 10 days of cold storage, it reached statistically similar results. The OP sample was mostly preferred to the OF sample, although the scores were very similar, indicating that oleaster peel flour makes a favorable difference in sensory properties and the commercialization chance of the oleaster puddings. Interestingly, some of the panelists noticed a slight sour taste and odor in oleaster puddings, but they highlighted an aromatic taste and odor on the tenth day of storage.

## 4 Conclusion

The results of the study showed that both peel and flesh flour of oleaster are potential ingredients for puddings and similar milk‐based desserts, improving the preferred features of the products such as antioxidant activity and nutritional quality. The oleaster enrichment causes a significant improvement in total phenolic content and antioxidant activity, adding value to the final product. The resulting puddings may favor the consumption of functional foods for people from all age groups, as being a novel alternative to the products on the market. Additional research could be made on different functional and health‐promoting activities of the developed products, including bioavailability tests, animal studies, and clinical trials. The potential value of other assays for antioxidant activity, other than DPPH, should also be evaluated in future studies.

## Author Contributions

**İrem Uzunsoy:** conceptualization, investigation, writing – original draft, methodology, validation, visualization, writing – review and editing, formal analysis, project administration, resources. **Elif Ayşe Anli:** investigation, writing – original draft, methodology, validation, visualization, writing – review and editing, formal analysis, resources. **Fundagül Erem:** conceptualization, investigation, writing – original draft, methodology, validation, visualization, writing – review and editing, formal analysis, project administration, resources.

## Funding

The authors have nothing to report.

## Ethics Statement

This study was approved by the Human Research Ethics Committee of Zonguldak Bülent Ecevit University.

## Consent

Written informed consent was obtained from all study participants.

## Conflicts of Interest

The authors declare no conflicts of interest.

## Supporting information


**Figure S1:** Images of commercial pudding samples and 3%, 5%, and 7% wheat‐flour containing primary experimental puddings.
**Figure S2:** Images of 5% oleaster flesh/peel flour containing primary experimental puddings, with 1%, 1.5%, and 2% of sodium alginate (SA).
**Figure S3:** Visual details of oleaster flour enriched pudding production, (a) Images of dry ingredients used in pudding samples, (b) Upper side view of pudding samples, (c) Front view of pudding samples.

## Data Availability

The data that support the findings of this study are available on request from the corresponding author. The data are not publicly available due to privacy or ethical restrictions.
