# Supplementary material for: Development of a Dairy Product Enriched With Oleaster Flour: A Novel Gluten‐Free Functional Pudding
Source: Food Sci Nutr. 2026 Aug 31;14(9):e72282. doi: 10.1002/fsn3.72282 (PMC13527734; doi:10.1002/fsn3.72282)
Supplement: Supplementary file 1 [file FSN3-14-e72282-s001.docx]

**SUPPLEMENTARY MATERIAL**

Fig. S1. Images of commercial pudding samples and 3, 5, and 7% wheat-flour containing primary experimental puddings.

Fig. S2. Images of 5% oleaster flesh/peel flour containing primary experimental puddings, with 1, 1.5, and 2% of sodium alginate (SA).

**C1	C2	OF	OP**

**b)**

c)

**C1	C2	OF	OP**

**c)**

Fig. S3. Visual details of oleaster flour enriched pudding production, a) Images of dry ingredients used in pudding samples, b) Upper side view of pudding samples, c) Front view of pudding samples.
